# Modulation of M1/M2 polarization by capsaicin contributes to the survival of dopaminergic neurons in the lipopolysaccharide-lesioned substantia nigra in vivo

**DOI:** 10.1038/s12276-018-0111-4

**Published:** 2018-07-03

**Authors:** Eugene Bok, Young Cheul Chung, Ki-Suk Kim, Hyung Hwan Baik, Won-Ho Shin, Byung Kwan Jin

**Affiliations:** 1 Predictive Model Research Center, Korea Institute of Toxicology, Daejeon, 34114 Korea; 2 Department of Biochemistry and Molecular Biology, School of Medicine Kyung Hee University, Seoul, 02447 Korea; 3 Department of Human and Environmental Toxicology, University of Science and Technology, Daejeon, 34113 Korea

**Keywords:** Neuroimmunology, Parkinson's disease

## Abstract

The present study examined the neuroprotective effects of capsaicin (CAP) and explored their underlying mechanisms in a lipopolysaccharide (LPS)-lesioned inflammatory rat model of Parkinson’s dieases (PD). LPS was unilaterally injected into the substantia nigra (SN) in the absence or presence of CAP or capsazepine (CZP, a TRPV1 antagonist). The SN tissues were prepared for immunohistochemical staining, reverse transcriptase-polymerase chain reaction (RT-PCR) analysis, western blot analysis, blood–brain barrier (BBB) permeability evaluation, and reactive oxygen species (ROS) detection. We found that CAP prevented the degeneration of nigral dopamine neurons in a dose-dependent manner and inhibited the expression of proinflammatory mediators in the LPS-lesioned SN. CAP shifted the proinflammatory M1 microglia/macrophage population to an anti-inflammatory M2 state as demonstrated by decreased expression of M1 markers (i.e., inducible nitric oxide synthase; iNOS and interleukin-6) and elevated expression of M2 markers (i.e., arginase 1 and CD206) in the SN. RT-PCR, western blotting, and immunohistochemical analysis demonstrated decreased iNOS expression and increased arginase 1 expression in the CAP-treated LPS-lesioned SN. Peroxynitrate production, reactive oxygen species levels and oxidative damage were reduced in the CAP-treated LPS-lesioned SN. The beneficial effects of CAP were blocked by CZP, indicating TRPV1 involvement. The present data indicate that CAP regulated the M1 and M2 activation states of microglia/macrophage in the LPS-lesioned SN, which resulted in the survival of dopamine neurons. It is therefore likely that TRPV1 activation by CAP has therapeutic potential for treating neurodegenerative diseases, that are associated with neuroinflammation and oxidative stress, such as PD.

## Parkinson’s disease: Preventing neuron death

A drug that activates a neuron-protecting protein in the brain may help treat Parkinson’s disease (PD). Scientists believe neurons die during PD because of an over-activation of proinflammatory markers within immune cell populations, such as the microglia and macrophage cells found in the central nervous system and the brain. Now, Byung Kwan Jin at Kyung Hee University in Seoul and Won-Ho Shin at the Korea Institute of Toxicology in Daejeon and co-workers have demonstrated that a proinflammatory state can be reversed in rat PD models by administering capsaicin, an analgesic drug. Capsaicin activates a receptor protein that is highly expressed in neurons, microglia and astrocytes, and may play a role in neuronal function and motor control. The protein activation reversed the inflammatory state of the immune cells, providing a more protective environment for neurons.

## Introduction

Parkinson’s disease (PD) is a common neurodegenerative disorder associated with progressive degeneration of dopamine (DA) neurons in the substantia nigra pars compacta (SNpc) and the loss of their fibers in the striatum (STR)^1^. Although the etiology of PD remains largely unknown^2^, accumulating evidence from human and animal studies suggest that PD is associated with inflammatory processes, such as microglial activation; infiltration of peripheral immune cells, including macrophages^3^, and blood–brain barrier (BBB) dysfunction^4^. Although several studies have provided evidence to distinguish between resident microglia and peripheral macrophages in the brain^5,6^, these two classes of cells are very similar in terms of their gene expression, cell surface markers and activation states^7^. Thus, those two cell types were combined and designated microglia/macrophages or CD11b^+^ cells.

Recent studies, including ours, have demonstrated that the activation of microglia/macrophages^8,9^ and the migration of peripheral T lymphocytes^8^ and neutrophils^10^ into the CNS are associated with the death of DA neurons in the SN. Increased BBB permeability and blood vessel changes are observed in the basal ganglia of patients with PD^11^ and play an important role in the death of DA neurons in lipopolysaccharide (LPS)-treated animal models of PD^12^.

Microglia/macrophage activation states are classified as the classical activation (or M1) state or the alternative activation (or M2) state. LPS, an agonist of the M1 state, induces the expression of cyclooxygenase-2 (COX-2), inducible nitric oxide synthase (iNOS), reactive oxygen species (ROS) such as nitric oxide (NO), and several proinflammatory cytokines including interleukin (IL)-1β. These are widely used as markers of the M1 state^13,14^ and elicit the degeneration of DA neurons in the SN^15,16^. By contrast, arginase 1 and CD206 (a mannose receptor), which are markers of the M2 state^17^ that localize in the inflammatory zone, block the expression of proinflammatory mediators such as iNOS^18^ and promote neuroprotection^19^.

The transient receptor potential vanilloid 1 (TRPV1) channel, which is a capsaicin (CAP) receptor, is involved in pain perception and is highly expressed in sensory neurons^20^. TRPV1 is also present in the brain, where it may play a role in modulating neuronal function^21^ and controlling motor behavior^22^. TRPV1 is activated by systemic administration of BBB-permeable CAP^23^. We previously demonstrated that TRPV1 activation by CAP protects mesencephalic DA neurons from 1-methyl-4-phenylpyridinium (MPP^+^) neurotoxicity by inhibiting microglia-derived oxidative stress^24^.

In the present study, we examined whether TRPV1 activation by CAP could regulate the M1/M2 state of microglia/macrophages in the LPS-lesioned SN, resulting in neuroprotection. We demonstrated that TRPV1 activation by CAP switched the M1/M2 state by increasing the expression of arginase 1 and CD206 (M2 markers) and decreasing iNOS, IL-1β, and IL-6 (M1 markers) in the LPS-lesioned SN, which led to the survival of DA neurons.

## Materials and methods

### Stereotaxic surgery

All experiments were performed in accordance with approved animal protocols and guidelines established by Kyung Hee University and the Institutional Animal Care and Use Committee of Korea Institute of Toxicology. As previously described^25^, Sprague Dawley (SD) rats (230–280 g) were anesthetized by injection of chloral hydrate (400 mg/kg, i.p.) and positioned in a stereotaxic apparatus. Each rat received a unilateral infusion of phosphate-buffered saline (PBS, as a control) or LPS into the right SN (anteroposterior, −5.2 mm; mediolateral, −2.1 mm; dorsoventral, −7.8 mm from bregma) according to the atlas of Paxinos and Watson (1998). All infusions were given using a Hamilton syringe equipped with a 30-gauge beveled needle and attached to a syringe pump (KD Scientific, MA, USA). Infusions were delivered at a rate of 0.5 μl/min for LPS (5 µg in 3 μl of sterile PBS; Sigma, MO, USA) and for PBS as controls. After injection, the needle was left in place for an additional 10 min before being slowly retracted. Animals were sacrificed and their brains harvested at the indicated time points.

### Pharmacological treatments

Capsaicin (CAP) was obtained from Sigma, and capsazepine (CZP) from Tocris (Ellisville, USA). Animals were treated with CAP (0.001–2.5 mg/kg, intraperitoneal (i.p.) injection) 30 min before LPS intranigral injection, and CZP (1 mg/kg, i.p.) was administered 30 min before CAP. Control animals received an intranigral injection of vehicle (PBS). CAP and CZP were dissolved in a vehicle consisting of an 8:1:1 ratio of PBS:TWEEN 80:absolute ethanol^26^. There was no difference in body weight or growth rate between any of the groups.

### Tissue preparation and immunohistochemistry

Brains tissues were prepared for immunohistochemical staining as previously described with some modifications^25,27^. In brief, animals were anesthetized with chloral hydrate (360 mg/kg, i.p.) at the indicated time points after stereotaxic surgery and transcardially perfused. Brains were frozen, sectioned into 40 µm coronal sections using a sliding microtome, and collected in six separate series. Immunohistochemistry was performed using the avidin-biotin staining technique. Free-floating serial sections were rinsed in PBS twice for 15 min and then pretreated for 5 min at room temperature (RT) in PBS containing 3% H_2_O_2_. The sections were then rinsed in PBS twice for 15 min each in PBS and blocked for 30 min at RT in PBS containing 5% normal serum (Vector Laboratories, Burlingame, CA, USA), 0.2% Triton X-100 (Sigma) and 1% BSA (Sigma). The sections were then rinsed in PBS containing 0.5% BSA twice for 15 min each. Next, the sections were incubated overnight with gentle shaking at RT in PBS containing 0.5% BSA and the following primary antibodies: mouse anti-neuron-specific nuclear protein (NeuN; 1:200; Merck Millipore, Temecula, CA) for neurons in general; anti-γgamma-aminobutyric acid (GABA; 1:1000; Sigma) for GABAergic neurons; rabbit anti-tyrosine hydroxylase (TH; 1:2000; Pel-Freez, Brown Deer, WI) for dopaminergic neurons; mouse anti-CD11b (1:500; Serotec, Oxford, UK), which recognizes complement receptor 3; mouse ED1 anti-CD68 (1:100; Serotec), a specific antibody against glycosylated lysosomal antigen for microglia/macrophages; rabbit anti-myeloperoxidase (MPO; 1:1000; DakoCytomation, Glostrup, Denmark), which recognized neutrophils; mouse anti-CD20 (1:1000; Thermo Fisher Scientific, Fremont, CA) which specifically recognizes B lymphocytes; rat anti-CD3 (1:1000, BD Pharmingen, San Diego, CA) which specifically recognizes T lymphocytes; mouse anti-OX-6 (1:200; BD Pharmingen), which specifically recognizes MHC class II; mouse anti-nitrotyrosine (1:50; Abcam, Cambridge, MA, USA), which recognizes NO-dependent oxidative stress; mouse anti-8-OHdG (1:300; Jaica, Shizuoka, Japan), which detects oxidative DNA damage; goat anti-IL-6 (1:200; Santa Cruz Biotechnology, Santa Cruz, CA); and rabbit anti-IL-10 (1:500; GeneTex, Irvine, CA, USA). The sections were then rinsed in PBS containing 0.5% BSA twice for 15 min each and incubated for 1 h at RT with biotin-conjugated anti-mouse (1:400; KPL, Gaithersburg, MD, USA), anti-rabbit (1:400; Vector Laboratories), or anti-goat secondary antibodies (1:200; Vector Laboratories). Sections were rinsed again in PBS containing 0.5% BSA and incubated for 1 h at RT in avidin-biotin complex (Vector Laboratories). After being rinsed twice in PB, the sections were incubated in 0.05% 3,3′ diaminobenzidine (Sigma) in 0.1 M PB containing 0.003% H_2_O_2_ to visualize the signal. Sections were then rinsed in 0.1 M PB, mounted on coated slides, and counterstained with hematoxylin (Merck Millipore). For Nissl staining, some of the SN tissue was stained with 0.1% cresyl violet (Sigma), dehydrated, and coverslipped. Stained tissues were analyzed under a bright-field microscope (Olympus Optical, Tokyo, Japan).

### Double immunofluorescence staining

For double immunofluorescence staining, tissue sections were processed as previously described with some modifications^27^. Briefly, free-floating sections were mounted on coated slides and dried for 30 min at RT. After being washed in PBS, sections were incubated in PBS containing 5% normal serum, 0.2% Triton X-100 and 1% BSA for 30 min and rinsed three times with PBS containing 0.5% BSA. The sections were incubated overnight at 4 °C in a combination of anti-IL-1β (1:200; IL-1β; R&D systems, Minneapolis, MN), anti-iNOS (diluted at 1:200; Upstate, Lake Placid, NY), anti-COX-2 (1:200; Santa Cruz Biotechnology), anti-arginase 1 (1:200; Santa Cruz Biotechnology), anti-CD206 (1:100; R&D system), IL-6 (1:200; Santa Cruz Biotechnology), or anti-IL-10 (1:500; GeneTex) and anti-CD11b. After being washed in PBS containing 0.5% BAS, the sections were incubated simultaneously with a mixture of FITC-conjugated chicken anti-mouse IgG (1:200, Molecular Probes, Eugene, OR) and Cy3-conjugated goat anti-rabbit IgG or Cy3-conjugated donkey anti-goat IgG (1:200; Molecular Probes) for 1 h at RT. Slides were coverslipped with Vectashield medium (Vector Laboratories) and viewed using an LSM 700 confocal laser scanning microscope (Carl Zeiss, Germany). To analyze the localization of different antigens in double-stained samples, we obtained images of different signals from the same area and merged them using interactive software.

### Stereological cell counting of DA neurons

As previously described^28,29^, the total number of TH-immunopositive neurons was counted in the various animal groups using the optical fractionator method performed on a bright-field microscope (Olympus Optical, BX51) using Stereo Investigator software (MBF Bioscience). This unbiased stereological method of cell counting is not affected by either the reference volume (SNpc) or the size of the counted elements (neurons).

### Counting of immunopositive cells

After sectioning the brains into 40 µm coronal sections using a sliding microtome and collecting them in six separate series, we chose one series and selected 3 evenly spaced sections from anterior to posterior in the SN region of the midbrain. The images were magnified by a factor of 100 (DAB staining) or 200 (immunofluorescence staining). Cell counts and evaluation of immunoreactivity were then conducted using Adobe Photoshop CS4. Every selected section passed through the SNpc region, containing up to 2.5 × 10^5^ μm^2^ of the SNpc for DAB staining or 1.2 × 10^5^ μm^2^ for immunofluorescence staining. Finally, immunopositive cells were counted using the “count tool” under the Analysis menu. The number of cells in each category was expressed as the percentage of the total population and/or the raw number.

### Fluorescein isothiocyanate (FITC)-labeled albumin Assay

As previously described^8^, a FITC-labeled albumin (Sigma) assay was performed for visualization of BBB leakage. After LPS or PBS injections, rats were transcardially perfused with Hanks’ Balanced Salt Solution containing heparin (10 U/ml), followed immediately by 10 ml FITC-labeled albumin (5 mg/ml, in 0.1 M PBS buffer) injected at a rate of 1.5 ml/min. Brains were dissected from the skull and postfixed overnight in buffered 4% PFA at 4 °C. After fixation, the brains were cut into 40 μm slices using a sliding microtome. Sections were mounted on gelatin-coated slides, and the vessels perfused with FITC-labeled albumin were examined by confocal microscopy (Carl Zeiss).

### Reverse transcription polymerase chain reaction (RT-PCR)

Brain tissues from the ipsilateral SN were dissected at the indicated time points after LPS or PBS injection with capsaicin or vehicle, and total RNA was extracted in a single step using RNAzol B (Tel-Test, Friendswood, TX) following the instructions of the manufacturer. Total RNA was reverse transcribed into cDNA using AMV reverse transcriptase (Promega, Madison, WI) and random primers (Promega). The primer sequences used in this study were as follows: 5′-TGATGTTCCCATTAGACAGC-3′ (forward) and 5′-GAGGTGCTGATGTAC CAG TT-3′ (reverse) for IL-1β (378 bp); 5′-ACACTCTATCACTGG CATCC-3′ (forward) and 5′-AAGGGACACCCTTTCACAT-3′ (reverse) for COX-2; 5′-GCAGAA TGTGACCATCATGG-3′ (forward) and 5′-ACAACCTTGGTGTTGAAGGC-3′ (reverse) for iNOS (557 bp); and 5′-TCCCTCAAGATTGTCAGCAA-3′ (forward) and 5′-AGATCCACAACGGATACATT-3′ (reverse) for glyceraldehyde-3-phosphate dehydrogenase (GAPDH; 308 bp). The PCR amplification consisted of 30 cycles of denaturation at 94 °C for 30 s, annealing at 50 °C for 30 s (for IL-1β and GAPDH or 54 °C for 30 s for iNOS) and extension at 72 °C for 90 s. PCR products were separated by electrophoresis on 1.5% agarose gels, after which the gels were stained with ethidium bromide and photographed. For semiquantitative analyses, the photographs were scanned using a Computer Imaging Device and the accompanying software (Fujifilm, Tokyo, Japan).

### Western blot analysis

For western blot analysis, tissue sections were processed as previously described with some modifications^29^. Brain tissues from the ipsilateral SN were dissected and homogenized in ice-cold lysis buffer containing the following (in mM): 150 NaCl, 10 Na_2_HPO_4_, PH 7.2, 0.5% sodium deoxycholate (deoxycholic acid, sodium salt C_24_H_39_NaO_4_), and 1% NP-40 plus protease inhibitor cocktail (Calbiochem, San Diego, CA, USA). The tissue homogenates were centrifuged at 4 °C for 20 min at 14,000 × *g*, and the supernatant was transferred to a fresh tube. The extracts were frozen and kept at −80 °C. Equal amounts of protein (50 μg) were loaded in each lane with loading buffer containing 0.125 M Tris-HCl (pH 6.8), pH 6.8, 20% glycerol, 4% SDS, 10% mercaptoethanol and 0.002% bromophenol blue. Samples were boiled for 5 min before being separated by SDS-PAGE. After electrophoresis, proteins were transferred to polyvinylidene difluoride membranes (Millipore) using an electrophoretic transfer system (Bio-Rad, Hercules, CA, USA). The membranes were washed with Tris-buffered saline solution containing 137 mM sodium chloride and 0.2% TWEEN 20 (TBST) and then blocked for 1 h in TBST containing 5% skim milk. The membranes were then incubated overnight at 4 °C with one of the following the specific primary antibodies: rabbit anti-iNOS (Millipore), mouse anti-COX-2 (BD Transduction) or mouse anti-arginase 1 (BD Transduction). After being washed, the membranes were incubated for 1 h at RT temperature with secondary antibodies (1:2000; Amersham Biosciences, Arlington Heights, IL) and washed again. Finally, the blots were developed with the ECL western blotting detection reagents (Amersham). The blots were re-probed with antibodies against mouse anti-actin (1:5000; Sigma). For semiquantitative analyses, the densities of bands on immunoblots were measured with a Computer Imaging Device and the accompanying software (Fujifilm).

### In situ detection of O_2_^−^ production

Numerous methods have been reported for in vivo detection of O_2_^−^ and O_2_^−^-derived oxidant production. Among these, we felt that hydroethidine conversion to ethidium might be a useful tool for detecting production of O_2_^−^ in our experiments because hydroethidine is selectively oxidized to ethidium by O_2_^−^, but not by other ROS^30^. For these experiments, hydroethidine (Molecular Probes; 1 mg/500 µl in 10% dimethyl sulfoxide with PBS) was intravenously administered 1 day after LPS injection, and the animals were killed 30 min later by transcardial perfusion. Brains were removed, postfixed, sectioned (40 μm), and mounted on coated slides. Ethidium accumulation, which represented generation of the oxidized hydroethidine products, was examined by confocal microscopy.

### Statistical analysis

All values are expressed as the mean ± SEM. Statistical significance (*p* < 0.05 for all analyses) was assessed by ANOVA using Instat 5.01 (GraphPad Software, San Diego, CA, USA), followed by Newman–Keuls or Bonferroni analyses. Statistical significance was defined as *p* < 0.05 for all analyses.

## Results

### Capsaicin prevents the degeneration of dopaminergic neurons in the lipopolysaccharide-injected substantia nigra in vivo

**Fig. 1:**
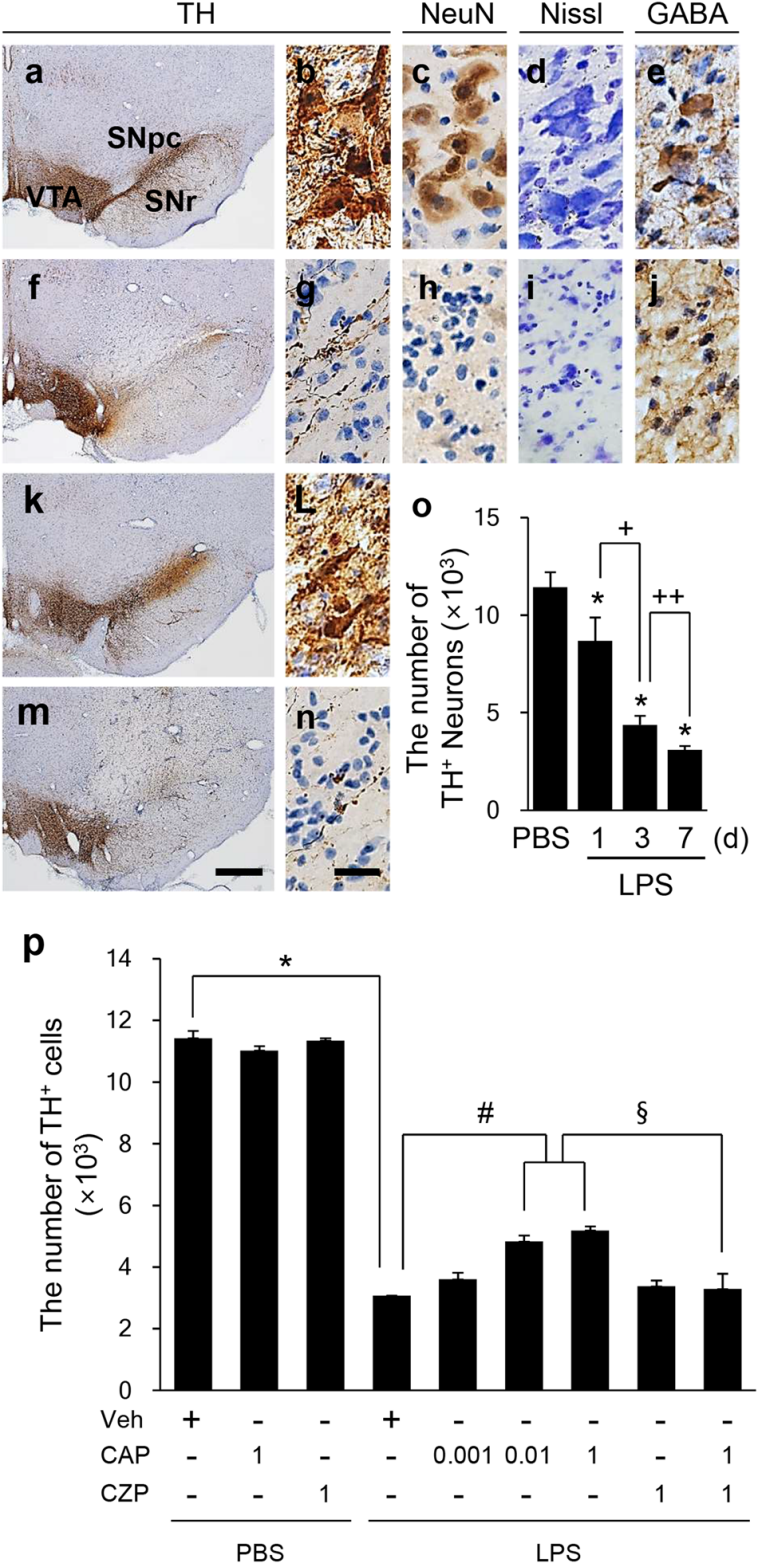
TRPV1 protected dopaminergic neurons in the SN from lipopolysaccharide (LPS)-induced neurotoxicity in vivo. Phosphate-buffered saline (PBS; **a**–**e**) or LPS (**f**–**n**, 5 µg/3 µl) was unilaterally injected into the SN in the absence (**f**–**j**) or presence (**k**–**n**) of capsaicin (CAP; 1 mg/kg, i.p.). CAP was administered 30 min before intranigral injection of PBS or LPS. Capsazepine (CZP; 1 mg/kg, i.p.; **m**–**n**) was administered 30 min before CAP. Animals were sacrificed after 7 days; brains were removed, and coronal sections (40 μm) were prepared using a sliding microtome. Every sixth serial section was selected and processed for TH (**a**, **b**, **f**, **g**, **k**–**n**), NeuN (**c**, **h**), or GABA immunostaining (**e**, **j**) or for Nissl staining (**d**, **i**). **b**, **g**, **l**, **n**, higher magnifications of **a**, **f**, **k**, and **m**, respectively. SNpc, substantia nigra pars compacta; SNr, substantia nigra reticulata; VTA, ventral tegmental area. Scale bars: **a**, **f**, **k**, **m**, 300 µm; **b**–**e**, **g**–**j**, **l**, **n**, 50 µm. **o**, **p**, the number of TH^+^ neurons in the whole SN was counted using a stereological technique at the indicated time points (**o**) and 7 days after LPS (**p**). The data are presented as the mean±SEM of 4 to 8 animals per group. *p < 0.001 compared with PBS-injected SN treated with vehicle; ^+^*p* < 0.001, ^++^*p* < 0.01 compared with LPS-injected SN; ^#^*p* < 0.001 compared with LPS-injected SN treated with vehicle; ^§^*p* < 0.001 compared with LPS-injected SN treated with CAP (ANOVA and Newman–Keuls method)

To examine the LPS-induced neurotoxicity in the SN, LPS (5 µg/3 µl) or PBS was unilaterally injected into the rat SN. The brains were then removed after 7 days, and sections were processed for Nissl staining, or immunostaining for NeuN or TH to detect neurons (in general) or DA neurons (specifically), respectively. In keeping with our recent results^25^, there was a significant loss of TH^+^ (Fig. 1f, g), NeuN^+^ (Fig. 1h), and Nissl-stained cells (Fig. 1i) in the LPS-treated SN compared with PBS-treated SN (Fig. 1a–d). Additional immunostaining also demonstrated the considerable loss of GABAergic neurons in the LPS-treated SN (Fig. 1j) compared with the PBS-treated SN (Fig. 1e). When TH^+^ neurons were quantified and expressed as a percentage of the quantity in the ipsilateral SN, we noted that LPS treatment decreased the number of TH^+^ neurons by 24% (Fig. 1o; *p* < 0.001), 61% (Fig. 1o; *p* < 0.001) and 73% (Fig. 1o; *p* < 0.001) at 1, 3, and 7 days after LPS, respectively, compared with the PBS control (Fig. 1o). By contrast, at 7 days after LPS, treatment with CAP, which is a TRPV1 agonist, significantly increased the number of TH^+^ neurons in the SN (Fig. 1k, l, p). Following the administration of 0.01 and 1 mg of CAP, 42 and 46% of TH^+^ neurons, respectively, remained from the total number of TH^+^ neurons in the SN (Fig. 1p; *p* < 0.001), which was significantly different compared with the percentage in the LPS-treated SN. The dose of 0.001 mg/kg of CAP did not have any significant effect. Neuroprotection by CAP was reversed by capsazepine (CZP; 1 mg/kg), which is a TRPV1 antagonist (Fig. 1m, n, p). This indicated that CAP elicited its neuroprotective effect via activation of TRPV1. The administration of vehicle, CAP (1 mg/kg), or CZP (1 mg/kg) alone had no effects on DA neurons in the SN (Fig. 1p).

### Capsaicin does not block lipopolysaccharide-induced infiltration of peripheral immune cells in the substantia nigra in vivo

**Fig. 2:**
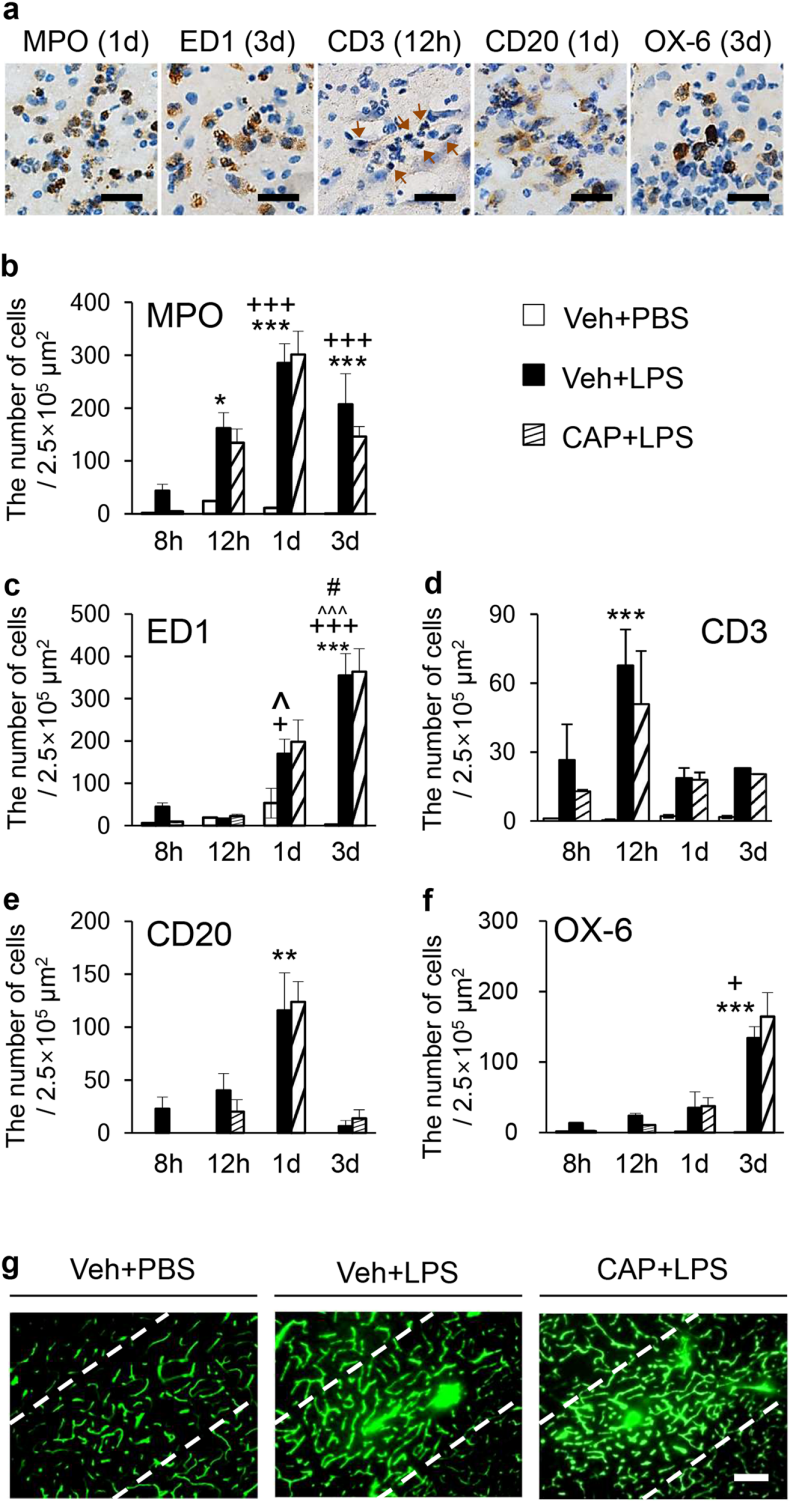
CAP failed to inhibit the infiltration of peripheral immune cells into the LPS-treated SN. **a** Every sixth serial section was selected and immunostained to identify expression of MPO, ED1, CD3, CD20, and OX-6 in the LPS (5 µg/3 µl)-treated SN at the indicated time points. Arrows indicate CD3^+^ cells. **b**–**f** The numbers of MPO^+^, ED1^+^, CD3^+^, CD20^+^, or OX-6^+^ cells in the SN. The data are presented as the mean±SEM of 4 to 5 animals per group. *p < 0.05, **p < 0.01, ***p < 0.001 compared with PBS-injected SN treated with vehicle; ^*+*^*p* < 0.05, ^*+++*^*p* < 0.001 compared with LPS-injected SN treated with vehicle at 8 h. ^^^*p* < 0.05, ^^^^^*p* < 0.001 compared with LPS-injected SN treated with vehicle at 12 h; ^*#*^*p* < 0.001, compared with LPS-injected SN treated with vehicle at 1 d (ANOVA and Bonferroni method). **g** A FITC-labeled albumin assay demonstrated that CAP did not prevent LPS-induced BBB disruption in the SN. The results represent 4–5 animals per group. Scale bar: **a** 25 µm; **g** 250 µm

Since LPS-induced infiltration of neutrophils and monocytes seems to be harmful for DA neurons in the SN^10^, we wondered whether an intranigral injection of LPS could produce infiltration of blood-borne peripheral immune cells, resulting in neuronal damage in the SN in vivo. Using antibodies against myeloperoxidase (MPO) to detect neutrophils, ED1 for phagocytic cells such as macrophage, CD3 for T cells, CD20 for B cells, and OX-6 for MHC II expressing cells, immunohistochemical analysis revealed a significant increase in infiltration of various types of peripheral immune cells in the LPS-treated SN, in a time-dependent manner, compared to the PBS-treated control. MPO^+^ cells were observed as early as 8 h after LPS, with maximal levels reached at 1 d after LPS and maintained up to 3 days after LPS (Fig. 2a, b). ED1^+^ cells were detected as early as 1 day after LPS and continuously increased up to 3 days after LPS (Fig. 2a, c). CD3^+^ (Fig. 2a, d) and CD20^+^ cells (Fig. 2a, e) were observed at 12 h and 1 day after LPS, which returned to basal levels at 1 d and 3 days after LPS, respectively. OX-6^+^ cells were observed at 3 days after LPS (Fig. 2a, f). The current results indicate that LPS induced the infiltration of peripheral immune cells in a time-dependent and/or cell-type-specific manner.

Thus, we hypothesized that CAP elicited neuroprotective effects by suppressing infiltration of potentially neurotoxic peripheral immune cells. However, immunostaining in the current study showed that CAP did not alter the infiltration of LPS-induced peripheral immune cells at any of the tested time points (Fig. 2b–f), which contradicts this hypothesis. Accordingly, we tested whether CAP affected LPS-induced BBB disruption. BBB disruption, which was measured using a FITC-labeled albumin assay, was detected in the SN 1 day after intranigral injection of PBS or LPS, in the absence or presence of CAP (1 mg/kg, i.p.) (Fig. 2g). In the PBS-treated SN (control), FITC-labeled albumin was confined to the blood vessels of the SN in vivo, indicating that the BBB was intact. By contrast, LPS produced considerable vascular permeability, which was not attenuated by CAP (Fig. 2g). These findings were in close agreement with results showing that CAP failed to reduce LPS-induced infiltration of peripheral immune cells.

### Capsaicin inhibits expression of lipopolysaccharide-induced proinflammatory mediators in the substantia nigra in vivo

**Fig. 3:**
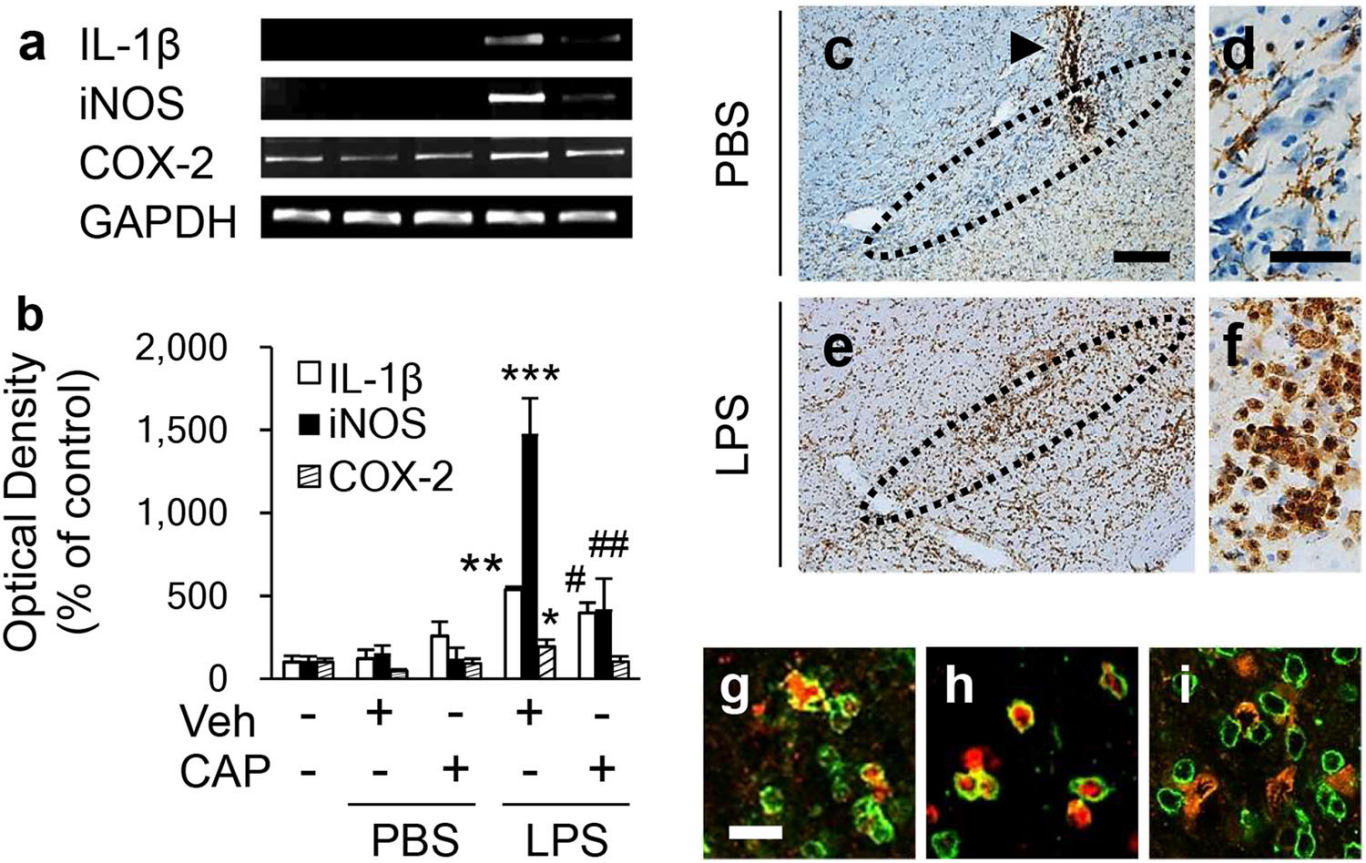
CAP reduced LPS-induced proinflammatory mediators in the SN. Animals were intranigrally infused with LPS (5 µg/3 µl) or PBS as a control in the presence or absence of CAP (1 mg/kg, i.p.) and killed 1 day later for RT-PCR (**a**, **b**) or immunohistochemical analysis (**c**-**i**) of the SN. **a** Total RNA isolated from the ipsilateral SN was analyzed by RT-PCR. Three to five animals were used for each experimental group. The data are presented as the mean ± SEM. ^*^p < 0.05, ^**^p < 0.01, ^***^p < 0.001 compared with PBS-injected SN treated with vehicle; ^#^*p* < 0.05, ^##^*p* < 0.001 compared with LPS-injected SN treated with vehicle (ANOVA and Newman–Keuls test). **c**–**f** The SN tissues were immunostained with CD11b in samples treated with PBS (**c**, **d**) or LPS (**e**, **f**). The data are representative of 6 to 8 animals for each experimental group. **d**, **f** Higher magnifications of **c** and **e**, respectively. Dotted lines indicate the substantia nigra pars compacta (SNpc). Arrowhead indicates the path of the needle. **g**–**i**, coexpression of IL-1β (red, **g**), iNOS (red, **h**), or COX-2 (red, **i**) with CD11b^+^ (green) in the LPS-treated SN at 1 day. Each image was captured from the same area and merged (yellow). Scale bar: **c**, **e**, 200 µm; **d**, **f**, 50 µm; **g**–**i**, 25 μm

Since CAP did not inhibit infiltration of peripheral immune cells or BBB damage in the LPS-treated SN in vivo, we investigated whether it could exert its neuroprotective effects by regulating the expression of various proinflammatory mediators. Using RT-PCR, we demonstrated that IL-1β, iNOS, and COX-2 mRNA level in the SN in vivo were increased at 1 d after LPS (Fig. 3a). CAP treatment significantly attenuated mRNA expression of IL-1β and iNOS but not COX-2 (Fig. 3a, b). Immunohistochemical staining was performed to determine the cellular localization of these markers. Consistent with our recent report^31^, the majority of CD11b^+^ cells exhibited a resting morphology (small cell bodies with thin, long or ramified processes) in PBS-treated SN (Fig. 3c, d). By contrast, the round CD11b^+^ cells were observed in the LPS-treated SN (Fig. 3e, f). Double immunofluorescence staining demonstrated that IL-1β, iNOS, and COX-2 were colocalized in CD11b^+^ cells in the LPS-treated SN (Fig. 3g–i). Additional immunohistochemical analysis showed that CAP induced no morphological changes of CD11b^+^ cells compared to control (vehicle) (Supplementary Fig. 1a). Similar to Fig. 3g–i, in CAP-treated LPS-lesioned SN, expression of IL-1β, iNOS and COX-2 protein in CD11b^+^ cells was also observed, although we did not quantify the data (Supplementary Fig. 1b).

### Capsaicin regulates M1 and M2 activation states of CD11b^+^ cells in the lipopolysaccharide-lesioned substantia nigra in vivo

**Fig. 4:**
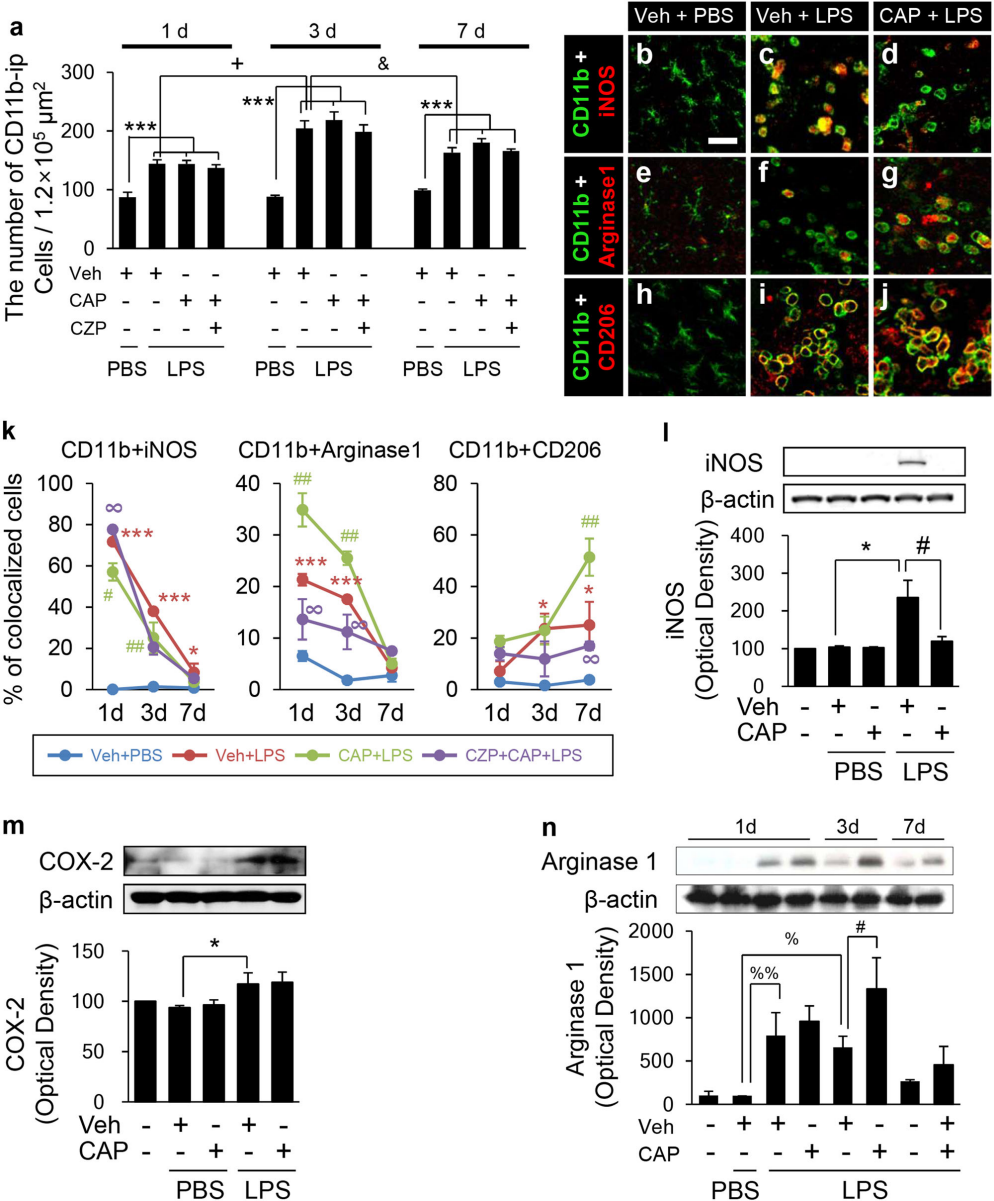
CAP regulated the M1 and M2 activation states. **a**–**j** SN tissue sections, adjacent to those used to produce Figs. 1 and 2, were immunostained with various antibodies. **a** The number of CD11b^+^ cells in the SN at the indicated time points. **b**–**j** SN sections were immunostained simultaneously with antibodies against CD11b and iNOS (**b**–**d**; 1 day), CD11b and arginase 1 (**e**–**g** 1 day), or CD11b and CD206 (**h**–**j**; 7 days). Each image was captured from the same area and merged (yellow). Scale bar: 25 μm. **k** Percentage of CD11b^+^ cells in the SN with iNOS^+^, arginase 1^+^, or CD206^+^ signal at the indicated time points. **l**–**n** Western blot analysis showing iNOS, COX-2, or arginase 1 expression in the LPS-treated SN, with or without CAP (1 mg/kg, i.p.). Intact or PBS-treated SN samples were used as controls. Optical density of bands for iNOS (**l**, 1 day), COX-2 (**m**, 1 day), or arginase 1 (**n**, at the indicated time points) were measured and quantified. The data are presented as the means±SEM of 4–5 animals per group. *p < 0.05, ***p < 0.001 compared with PBS-injected SN treated with vehicle at the same time point; ^#^*p* < 0.05, ^##^*p* < 0.01 compared with LPS-injected SN treated with vehicle at the same time point; ^∞^*p* < 0.01 compared with LPS-injected SN treated with CAP at the same time point; ^+^*p* < 0.001 compared with LPS-injected SN treated with vehicle at 1 d; ^&^*p* < 0.01, compared with LPS-injected SN treated with vehicle at 3 days; ^%^*p* < 0.05, ^%%^*p* < 0.01 compared with PBS-injected SN treated with vehicle (ANOVA and Bonferroni method)

Similar to the data shown in Fig. 2, immunohistochemical analysis confirmed that treatment with CAP did not reduce the LPS-induced increase in CD11b^+^ cells in the SN at any of the time points tested here (Fig. 4a). Since CAP seemed to regulate M1 activation markers (IL-1β and iNOS) in the SN in vivo (Fig. 3), we hypothesized that it could exert neuroprotection by regulating the M1 and M2 activation state^32^. Thus, we analyzed the effects of CAP on iNOS and arginase 1 expression as an M1 and M2 marker, respectively. Immunohistochemical analysis revealed iNOS expression reached by 71% (*P* < 0.001) and 38% (*P* < 0.001) at 1 and 3 days after LPS, respectively, in CD11b^+^ cells in the SN (Fig. 4b, c, k). CAP attenuated the LPS-induced increase in iNOS expression by 14% (*P* < 0.05), at both 1 day and 3 days after LPS (Fig. 4d, k). These CAP effects were reversed by CZP (Fig. 4k). As an M2 activation state marker, arginase 1 was increased by 21% (*P* < 0.001) and 17% (*P* < 0.001) at 1 and 3 days after LPS, respectively, in CD11b^+^ cells in the SN (Fig. 4e, f, k). Further, CAP significantly enhanced the LPS-induced increase in arginase 1 expression by 11% (*P* < 0.01) and 8% (*P* < 0.05) at 1 and 3 days after LPS, respectively (Fig. 4g, k), which were reversed by CZP (Fig. 4k). As another M2 activation state marker, CD206 was also expressed by 23% (*P* < 0.05) and 22% (*p* < 0.05) at 3 and 7 days after LPS, respectively (Fig. 4h, i, k). Similar to arginase 1, CAP significantly enhanced the LPS-induced increase in CD206 expression by 26% (*P* < 0.01) at 7 d after LPS (Fig. 4j, k), which were reversed by CZP (Fig. 4k).

As LPS-induced expression of IL-6, an M1 marker, in microglia contributes to degeneration of DA neurons^33^ and LPS-induced expression of IL-10, an M2 marker, in microglia contributed to neuronal survival^34^, we wondered if CAP could regulate the expression of IL-6 or IL-10 in CD11b^+^ cells. Immunohistochemical analysis revealed that in LPS-lesioned SN, both IL-6 and IL-10 were highly expressed at 3 days after LPS compared to PBS control SN (Supplementary Fig. 2a). Double immunohistochemical analysis exhibited a significant increase in expression of IL-6 by 39% (*P* < 0.001; Supplementary Fig. 2b, c) and IL-10 by 42% (*P* < 0.001; Supplementary Fig. 2d, e) in CD11b^+^ cells. In CAP-treated LPS-lesioned SN, IL-6 expression in CD11b^+^ cells was significantly decreased by 18% (*P* < 0.05; Supplementary Fig. 2b, c) with unchanged levels of IL-10 expression (Supplementary Fig. 2d, e).

Similar to immunohistochemical and RT-PCR data, western blot analysis demonstrated that LPS enhanced the expression of iNOS and COX-2 protein in the SN at 1 day after LPS compared with the PBS control. In CAP-treated LPS-lesioned SN, the protein levels of iNOS, but not COX-2, significantly decreased compared with LPS-lesioned SN (Fig. 4l, m) which was comparable to the mRNA expression shown in Fig. 3. The protein level of arginase 1 peaked at 1 d and returned to normal at 7 days (Fig. 4n). Further, CAP treatment significantly enhanced the expression of arginase 1, at 3 and 7 days after LPS. These data indicated that, in the LPS-lesioned SN in vivo, CAP regulated iNOS expression in an inhibitory manner, which was opposite to its regulation of arginase 1 expression.

The phenotypic changes of microglia/macrophages in vivo could have been a direct result of CAP injection or could have been due to CAP-activated reactive astrocytes, which are activated by CAP^35^. To investigate this, we exposed astrocyte-free primary rat microglia cultures to LPS in the absence or presence of CAP. Consistent with in vivo data, western blot analysis revealed that CAP significantly decreased iNOS expression and increased arginase 1 expression, in LPS-treated cortical microglia (Supplementary Fig. 3a–c). Additionally, CAP also attenuated LPS-induced NO release in cortical and mesencephalic microglia cultures (Supplementary Fig. 3d), indicating a direct effect of CAP.

### Capsaicin inhibits lipopolysaccharide-induced peroxynitrate production and oxidative stress in the substantia nigra

**Fig. 5:**
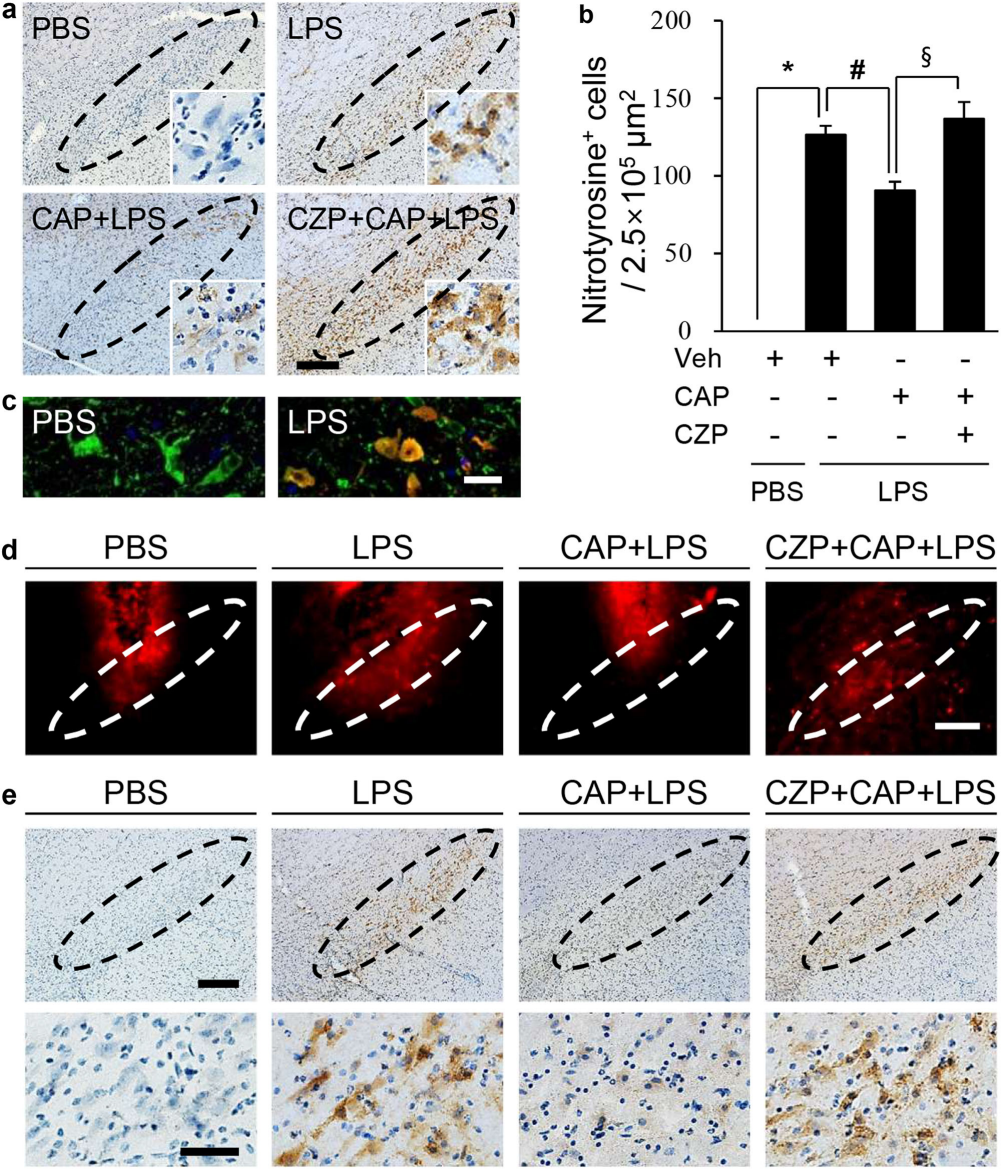
CAP inhibited LPS-induced peroxynitrate production and oxidative stress in the SN in vivo. Animals received a unilateral injection of PBS or LPS (5 µg/3 µl) into the SN in the absence or presence of CAP (1 mg/kg, i.p.). Animals were then transcardially perfused for further analysis at 1 day after LPS. CAP was administered 30 min before intranigral injection of PBS or LPS; CZP (1 mg/kg, i.p.) was administered 30 min before CAP. **a** Photomicrograph of nitrotyrosine^+^ cells. Dotted lines indicate the SN pars compacta (SNpc). Insets show highly magnified nitrotyrosine^+^ cells. **b** Number of nitrotyrosine^+^ cells in the SNpc. The data are presented as the mean ± SEM of 3–5 animals per group. **p < 0.001 compared with PBS-injected SN; ^#^*p* < 0.05 compared with LPS-injected SN (ANOVA and Student–Newman–Keuls method). **c** Fluorescence images of nitrotyrosine (red) and TH (green) separately and their merged signals (yellow) in the SNpc. **d** In situ visualization of LPS-induced O_2_^-^ and O_2_^-^-derived oxidant production in the absence or presence of CAP (1 mg/kg, i.p.) or CAP with CZP in the SN. Confocal micrographs show ethidium fluorescence (red), and the dotted lines indicate the SNpc, which showed degeneration of dopaminergic neurons after LPS treatment. **e** SN tissues adjacent to those used in **a**–**c** were prepared for 8-OHdG histochemistry to detect oxidative DNA damage in the SN. Dotted lines indicate the SNpc. Lower panels show highly magnified 8-OHdG^+^ cells. Scale bars: **a, d, e** (upper), 200 µm; **c**, 25 µm; **e** (lower), 50 µm. The results represent 3 to 4 animals per group

Nitration of protein tyrosine residues, a well-known marker of oxidative stress in patients with PD^36^ and in animal models of PD^37^, is mediated by iNOS-derived oxidants. As CAP attenuated LPS-induced iNOS expression and NO production (Figs 3, 4 and Supplementary Fig. 1), we investigated if it could alter the level of nitration in vivo in the LPS-lesioned SN. Immunohistochemical analysis demonstrated the significant increase in nitrotyrosine^+^ cells in the LPS-treated SN (Fig. 5a, b), compared with control (Fig. 5a, b). This effect was significantly attenuated by CAP, and the effect of CAP was subsequently reversed by CZP (Fig. 5a, b). Double immunofluorescence staining indicated that the majority of nitrotyrosine^+^ cells were colocalized in TH^+^ DA neurons in the LPS-lesioned SN, compared to the PBS control SN (Fig. 5c), suggesting that CAP protects DA neurons from nitrosative damage.

Activated microglia can produce O_2_^−^ and O_2_^−^-derived oxidants, which contribute to DA neuronal death in the SN^25^. Accordingly, we examined whether CAP could rescue nigral DA neurons by inhibiting LPS-induced oxidant production. To test this, hydroethidine histochemistry was performed at 1 day after LPS for in situ visualization of O_2_^−^ production. The accumulation of fluorescent products of oxidized hydroethidine (i.e., ethidium) significantly increased at 1 d after LPS in the LPS-lesioned SN, compared with the control (Fig. 5d). CAP diminished ethidium accumulation in the LPS-lesioned SN, which was reversed by CZP (Fig. 5d).

We recently demonstrated that DA neuronal cell death was accompanied by increased levels of 8-OHdG, a marker of oxidative nucleic acid damage^37,38^. Immunostaining demonstrated a significant increase in the levels of 8-OHdG in the LPS-lesioned SN compared to the control (Fig. 5e). CAP, However, CAP dramatically reduced 8-OHdG levels in the LPS-lesioned SN, which was reversed by CZP (Fig. 5e). These results suggested that CAP prevented LPS-induced oxidative damage to DNA in the SN.

## Discussion

The present study demonstrates that CAP might regulate M1 and M2 polarization in microglia/macrophages, subsequently rescuing DA neurons in the LPS-lesioned SN. CZP partially inhibited the neuroprotective effects of CAP, indicating TRPV1 involvement.

It seems noteworthy that although the M1/M2 paradigm might be inappropriate for strict application to microglia^39^, the M1/M2 paradigm is still a useful framework for studying not only peripheral macrophages^40^ but also brain microglia^41,42^ under inflammatory conditions.

Microglia/macrophage become polarized towards a proinflammatory (M1) phenotype^17,43^ upon various forms of stimulation, including LPS, and subsequently produce proinflammatory cytokines, such as IL-1β, IL-6 and ROS^44^.

Mounting evidence has demonstrated microglial activation and upregulation of IL-1β in the SN of patients with PD^45^ and LPS-lesioned SN^9^. Recent studies indicated that CAP treatment attenuated MPP^+^-induced microglial activation in the SN in vivo^24^ and lowered the kainic acid-induced increase in IL-1β in the rat hippocampus, which subsequently protected neurons in these regions during the insult^46^. IL-1β was found to mediate DA neuronal death in the SN. The current study demonstrated that treatment with CAP inhibited the LPS-induced elevation in IL-1β mRNA expression.

Additionally, LPS-induced expression of IL-6, an M1 marker, in microglia contributes to degeneration of DA neurons^33^ and elevation of IL-6 in patients with PD contributes to mortality risk^47,48^, indicating IL-6 neurotoxicity. This is in line with our data showing that CAP reduces LPS-induced IL-6 expression in CD11b^+^ cells, resulting in neuroprotection. Collectively, the observed CAP neuroprotection was associated with its ability to inhibit microglial activation and the expression of proinflammatory cytokines, such as IL-1β and IL-6, markers of the M1 phenotype.

By contrast, CAP failed to reduce the expression level of the LPS-induced COX-2 protein or mRNA. The result is similar to the previous report that CAP did not affect the COX-2 expression at either the protein or mRNA level, but inhibited the enzyme activity of COX-2 and the expression of the iNOS protein in LPS-stimulated peritoneal macrophages^49^. It is therefore likely that CAP selectively regulates M1/M2 phenotypes depending on the experimental conditions.

ROS, such as O_2_^−^ and O_2_^−^-derived oxidants, induce degeneration of DA neurons in the SN^50^. In the LPS-induced inflammation model, ROS can cause loss of DA neurons in the SN by producing oxidative damage to proteins^31^. In the 1-methyl-4-phenyl-1,2,3,6-tetrahydropyridine (MPTP) model of PD, ROS, which were generated by activated microglia, triggered oxidative damage to DNA, leading to DA neuronal death^37,51^. Many postmortem studies have also demonstrated oxidative damage to proteins, lipids, and DNA in PD^52^. The results of the present study showed LPS-increased extracellular ROS and DNA damage in the SN. CAP treatment, however, not only inhibited ROS production but also mitigated DNA oxidation. Collectively, the data suggest that CAP prevents LPS-induced ROS production and oxidative damage, resulting in neuroprotection.

In addition to ROS, LPS-activated M1 microglia/macrophages also highly upregulate the expression of iNOS for NO production and nitrotyrosine^9^, which impose oxidative damage on DA neurons in the SN. iNOS expression is increased in the brains of patients with PD and representative of microglia/macrophage in the M1 phase^43^. NO, generated by iNOS, may also play a role in the pathogenesis of PD^53^. NO^−^-mediated neurotoxicity is attributed to its reaction with superoxide, which subsequently produces peroxynitrate (ONOO^-^) that causes oxidative stress to proteins, via the nitration of tyrosine residues, subsequently leading to the degeneration of DA neurons in 6-hydroxydopamine, LPS and MPTP models of PD^37,54^. In the present study, CAP reduced iNOS expression and decreased levels of TH nitrotyrosine in the LPS-lesioned rat SN. These data suggest that CAP attenuated LPS-induced iNOS expression (indicative of the M1 phenotype) and/or oxidative stress in DA neurons, resulting in neuroprotection.

Microglia/macrophage activation and M1/M2 polarization have been well characterized in several types of acute central nervous system injury, including intracerebral hemorrhage injury^55^, ischemic stroke^56^, spinal cord injury^19^ and traumatic brain injury^57^. Conversion of M1 to M2 microglia/macrophage prevented degeneration of DA neurons in the MPTP mouse model of PD^58^. Fasudil, a Rho kinase inhibitor, increased the expression of arginase 1 (an M2 marker) and decreased the expression of iNOS (an M1 marker), both of which were expressed in CD11b^+^ cells in the MPTP-treated mouse SN. Thus, fasudil was neuroprotective and promoted recovery of motor function^58^. There are several reports showing that arginase 1 activation limited the availability of arginine as a substrate for iNOS, thereby negatively regulating its enzymatic activity^59^. D206, another M2 marker can bind to apoptotic and necrotic cells, facilitating the removal of dying cells, without causing secondary damage^58^. In the injured mouse spinal cord, both arginase 1 and CD206 produced by M2 macrophages promoted axonal growth^19^. In the present study, both M2 phenotypic markers were upregulated; arginase 1 and CD206 were co-expressed in CD11b^+^ cells more frequently in CAP-treated LPS-lesioned rat SN than in vehicle-treated LPS-lesioned rat SN. CAP regulates phenotypes of macrophages by increasing IL-10 expression^60^, i.e., another important M2 mediator^61^ and neuroprotective cytokine^34^. However, CAP treatment did not alter the expression of IL-10 in CD11b^+^ cells in LPS-lesioned SN. The neuroprotective effect of CAP was reduced by pharmacological inhibition (CZP), indicating that this effect was elicited via TRPV1 activation. Collectively, these data suggest that selective modulation of CAP on M1/M2 polarization of microglia/macrophage might be involved in TRPV1-dependent neuroprotective mechanism of CAP.

Moreover, CAP directly modulated NO production in LPS-treated primary cultured mesencephalic microglia (Supplementary Fig. 1). On the basis of the combined evidence, we tentatively suggest that TRPV1 activation by CAP contributed to neuroprotection by the functional conversion of microglia/macrophages from M1 to M2.

Damage to the BBB is also closely associated with the death of DA neurons in the SN. BBB disruption is observed in the basal ganglia of patients with PD^11^ and can enhance degeneration of DA neurons in the LPS-treated rat SN^12^ and in the MPTP-treated mouse SN^8^. Accumulating evidence, including ours, has demonstrated that infiltration of peripheral immune cells, such as neutrophils, T cells, B cells, macrophages and OX-6^+^ cells are involved in DA neuronal death in the LPS-lesioned rat SN^10,62^, in the MPTP-treated mouse SN^8^, and in the SN of patients with PD^63,64^. However, the data obtained from the current study demonstrated that CAP failed to inhibit increased BBB disruption and infiltration of peripheral neurotoxic immune cells into the LPS-lesioned SN. It is therefore likely that CAP elicits its neuroprotective effect by the M1/M2 functional conversion of microglia/macrophages without affecting the BBB or infiltration of peripheral immune cells into the SN.

Our work reveals a new insight into CAP neuroprotection. CAP may regulate the temporal expression of M1- and M_2_-related factors by shifting the state of microglia/macrophages from M1 to M2 in the LPS-lesioned SN, resulting in survival of DA neurons. The effects of CAP are inhibited by CZP, indicating the involvement of TRPV1. Taken together, the present data suggest that TRPV1 activation by CAP or related compounds might be beneficial for treating neurodegenerative diseases associated with neuroinflammation, such as PD.

## Electronic supplementary material

Supplementary Figure 1

Supplementary Figure 2

Supplementary Figure 3

SUPPLEMENTAL MATERIAL
