# Supplementary figures and images for: Modulation of M1/M2 polarization by capsaicin contributes to the survival of dopaminergic neurons in the lipopolysaccharide-lesioned substantia nigra in vivo
Source: Exp Mol Med. 2018 Jul 3;50(7):1–14. doi: 10.1038/s12276-018-0111-4 (PMC6030094; doi:10.1038/s12276-018-0111-4)

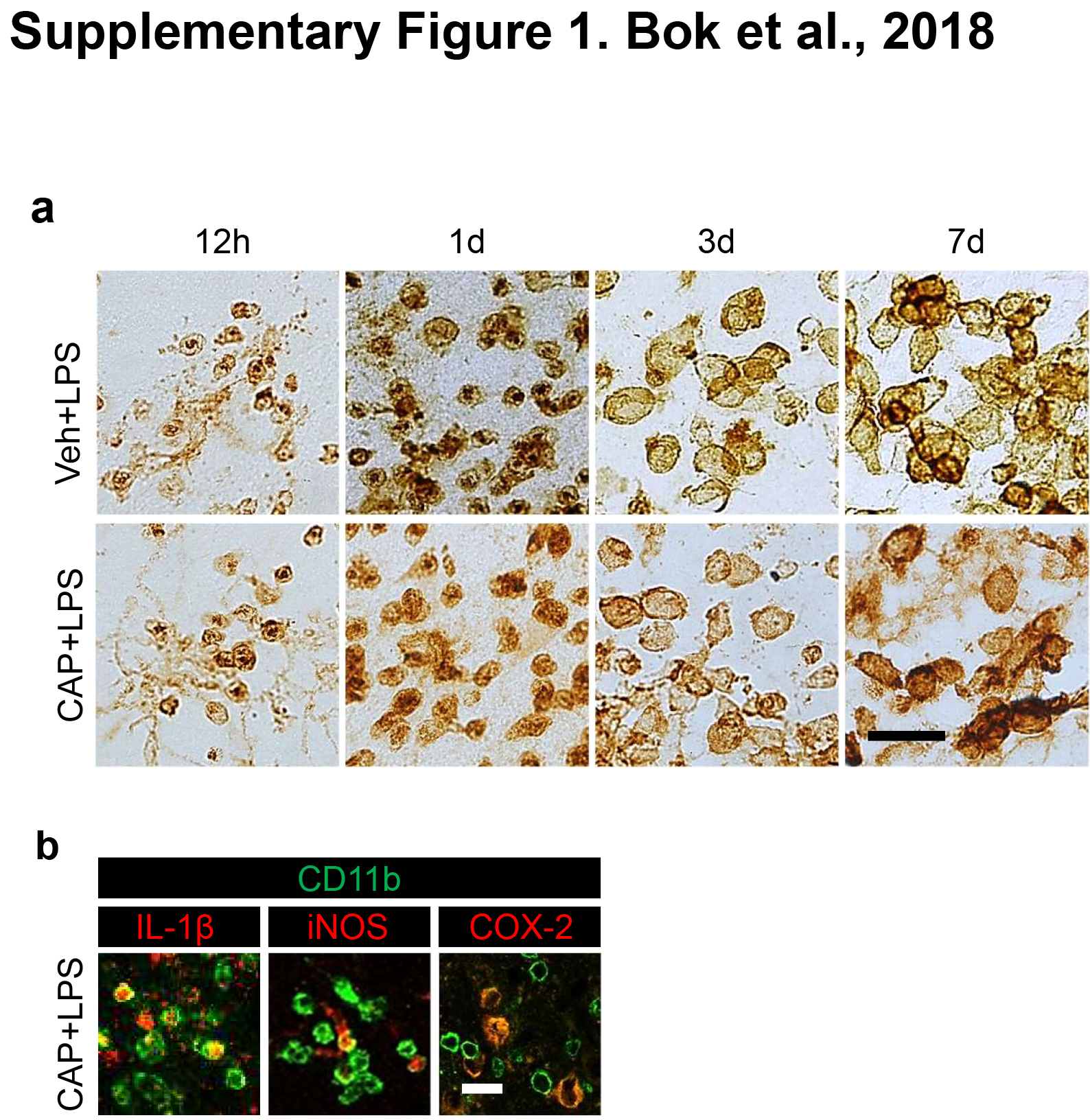

Supplement: Supplementary file 1 — Supplementary Figure 1 [file 12276_2018_111_MOESM1_ESM.tif]

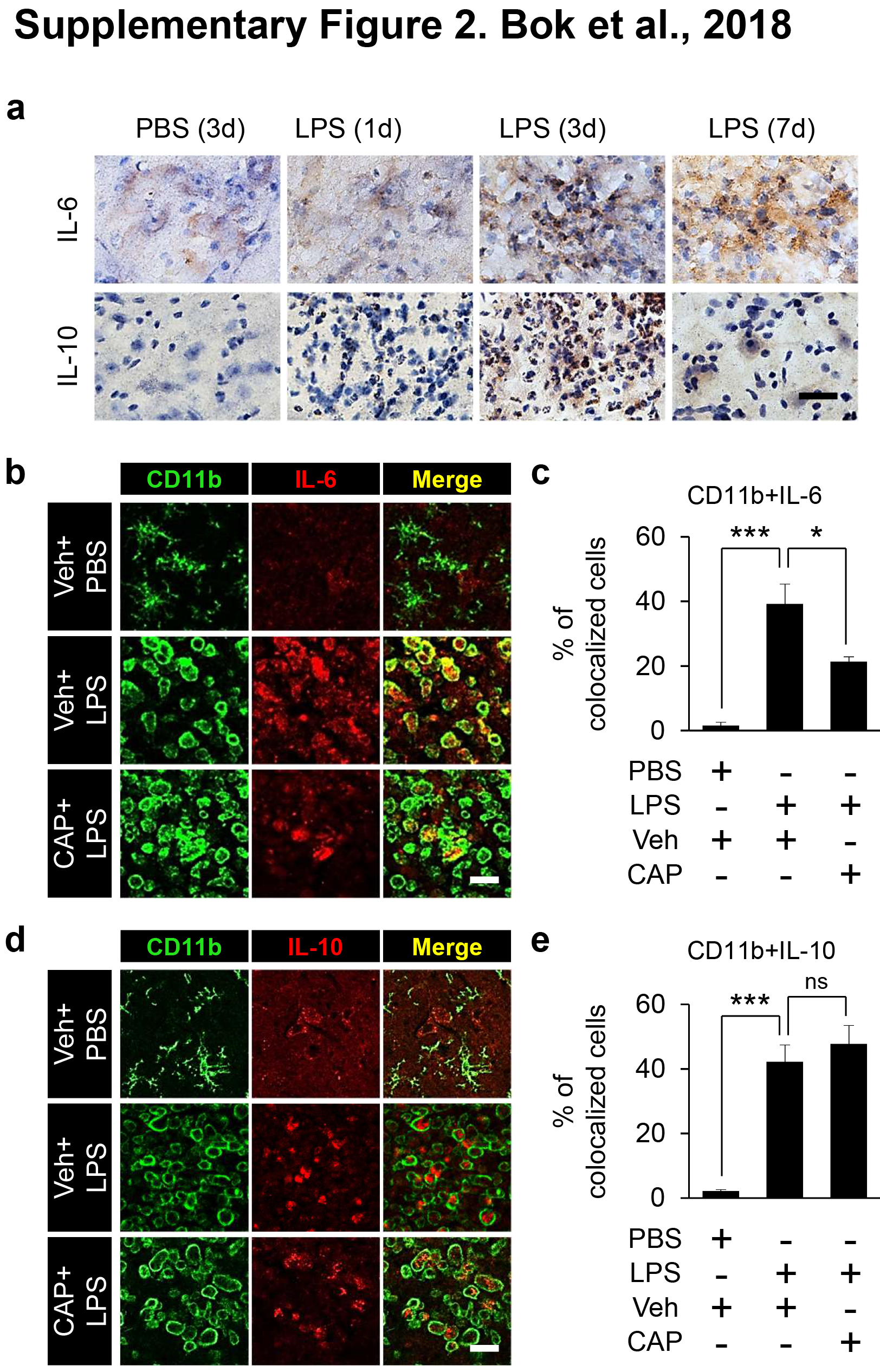

Supplement: Supplementary file 2 — Supplementary Figure 2 [file 12276_2018_111_MOESM2_ESM.tif]

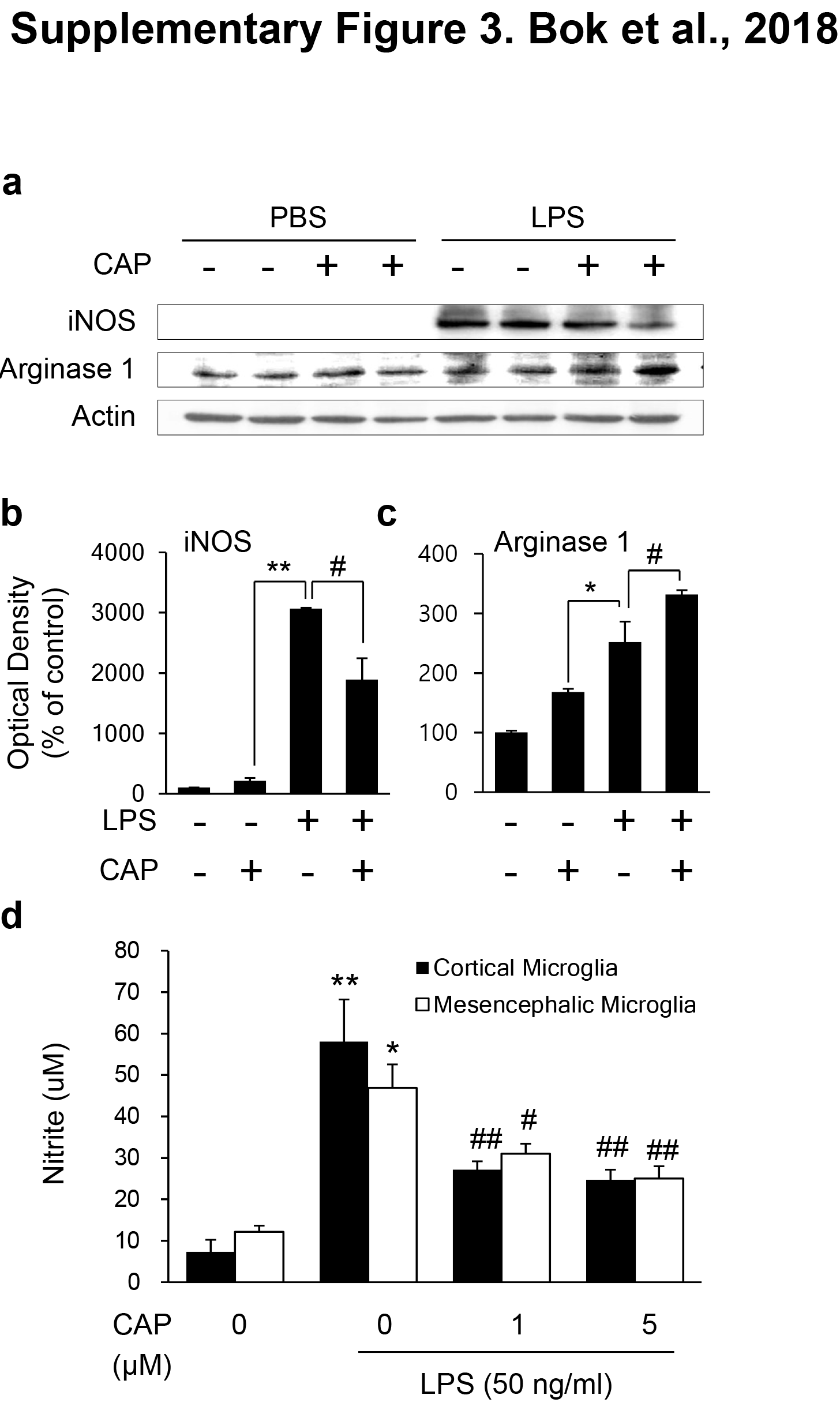

Supplement: Supplementary file 3 — Supplementary Figure 3 [file 12276_2018_111_MOESM3_ESM.tif]
