# Supplementary material for: Modulation of M1/M2 polarization by capsaicin contributes to the survival of dopaminergic neurons in the lipopolysaccharide-lesioned substantia nigra in vivo
Source: Exp Mol Med. 2018 Jul 3;50(7):1–14. doi: 10.1038/s12276-018-0111-4 (PMC6030094; doi:10.1038/s12276-018-0111-4)
Supplement: Supplementary file 4 — SUPPLEMENTAL MATERIAL [file 12276_2018_111_MOESM4_ESM.doc]

**Supplementary Materials and Methods**

*Cortical and mesencephalic microglia cultures*

As previously described [^1^](#_ENREF_1), For cortical microglia, microglia were obtained from the cerebral cortices of postnatal day 1 SD rats, and meninges and blood vessels were removed. Cortices were minced, and cells were dissociated at 25 ℃ in Leibovitz’s L-15 media. Digestion was terminated by adding and equal volume of MEM containing 10 % fetal bovine serum (FBS; Gibco, Rockville, MD, USA), and cells were triturated to obtain a single cell suspension. Cells were grown for 2 weeks in 75 cm^2^ T-flasks. For mesencephalic microglia cultures, microglia were obtained from the ventral mesencephalons of embryonic day 14 SD rats. Tissues were cut into small pieces and incubated in Ca^2+^, Mg^2+^ free Hanks' balanced salt solution (CMF-HBSS) for 10 min at 37 ℃ in a rotator (40 r.p.m.). Cultures were replaced with a 0.01% trypsin solution (in CMF-HBSS), incubated for an additional 10 min, then rinsed twice in RF medium containing Dulbecco's modified Eagle's medium (DMEM), 10% fetal bovine serum, glucose (6 mg/ml), L-glutamine (204 μg/ml), and penicillin/streptomycin (P/S, 100 U/ml), and mechanically triturated. Dissociated cells were seeded 75 cm^2^ T-flasks. After 2±3 weeks, microglia were detached from the flasks by mild shaking and filtered through a nylon mesh to remove astrocytes and then plated into 24 well plates at a density of 5×10^4^ cells/well or 35 mm culture dishes (5×10^5^ cells per well). 1 hour later, the culture medium was changed to RF medium containing 2% fetal bovine serum. After 24 hours, cells were treated with LPS (50 ng/ml) or with LPS and CAP for the 48 hours to obtain conditioned media. CAP (1 ~ 5 µM) were treated 30 min before LPS administration. Microglia were then processed for measurement of nitrite formation or western blot analysis.

*Determination of NO*

For measuring NO release, cultures of cortical and mesencephalic microglia were treated with LPS 48 hours. As described previously [^2^](#_ENREF_2), the amount of nitrite converted from NO was measured by mixing the culture media (50 ul) with an equal volume of Griess reagent (0.1% naphthylethylene diamine, 1% sulfanylamide, 2.5% H3PO4). The optical densities were measured at 540 nm.

*Image J Anlaysis*

Fluorescence stained samples were analyzed under confocal laser-scanning microscope (Carl Zeiss). To analyze the localization of different antigens in double-stained samples, images were obtained from the same area and merged using interactive software. Imaging data were analyzed in Image J (National Institutes of Health) as described recently [^3^](#_ENREF_3). ImageJ with co-localization plugin was used to quantify immunofluorescence and with colour deconvolution plugin was used to quantify chromogenic signal intensity on image.

**Supplementary Reference**

1. Chung ES, Joe EH, Ryu JK, Kim J, Lee YB, Cho KG *et al.* GT1b ganglioside induces death of dopaminergic neurons in rat mesencephalic cultures. *Neuroreport* 2001; **12:** 611-614.

2. Lee DY, Oh YJ, Jin BK. Thrombin-activated microglia contribute to death of dopaminergic neurons in rat mesencephalic cultures: dual roles of mitogen-activated protein kinase signaling pathways. *Glia* 2005; **51:** 98-110.

3. Nam JH, Park ES, Won SY, Lee YA, Kim KI, Jeong JY *et al.* TRPV1 on astrocytes rescues nigral dopamine neurons in Parkinson's disease via CNTF. *Brain* 2015; **138:** 3610-3622.

**Supplementary Figure legends**

**Supplementary figure 1.** Immunohistochemical staining of CD11b^+^ cells in the LPS-injected SN *in vivo*. Animals were intranigrally received LPS (5 µg/3 µl) or PBS as a control in the absence or presence of CAP (1 mg/kg, i.p.) and sacrificed at indicated time points. SN tissues were prepared for immunostaining. **a,** Photomicrographs of CD11b^+^ cells in the SN. **b,** Colocalization of IL-1β (red), iNOS (red), or COX-2 (red), within CD11b^+^ cells (green) in the LPS-lesioned SN in the presence of CAP at 1 d post LPS. Each image was captured from the same area and merged (yellow). The data are representative of 5 to 6 animals used for each experimental group. Scale bar: a, b, 25 μm.

**Supplementary figure 2.** LPS induces expression of IL-6 and IL-10 in the SN *in vivo*. SN tissue sections adjacent to those used in Supplementary Figure 1, were immunostained with antibodies against IL-6 or IL-10. **a**, Photomicrographs of IL-6^+^ and IL-10^+^ cells in the SN *in vivo*. **b-e**, Colocalization (b, d) and quantification (c, e) of IL-6 (red; b, c) or IL-10 (red; d, e) within CD11b^+^ cells (green) in the CAP-treated LPS-injected SN at 3 d post LPS. Each image was captured from the same area and merged (yellow). Data presented as means ± SEM of 4 animals per group. **p*<0.05, ****p*<0.001 (ANOVA and Newman-Keuls analyses). Scale bar: a, b, d, 25 µm.

**Supplementary figure 3.** CAP attenuates LPS-induced expression of iNOS and arginase 1, and releasing of NO in cultured rat microglia. **a**, western blot analysis shows expression of iNOS or Arginase 1 in cultured cortical microglia exposed to LPS (50 ng/ml) or PBS as a control for 1 d in the absence or presence of CAP (1 uM). The optical density of the bands for iNOS (**b**) and arginase 1 (**c**) were measured and quantified. Data were presented as mean ± SEM of duplicate cultures in three separate samples. *p<0.05, ^**^*p*<0.001, compared with non-treated microglia, ^#^*p*<0.05, compared with LPS-treated microglia, (ANOVA and Student-Newman-Keuls analyses). **d**, Cortical or mesencephalic microglia cultures were treated with LPS for 2 d. Nitrite content in the supernatant was then measured. Results are representative of 3 to 4 independent replicate experiments. Data presented as means ± SEM; *p<0.01, ***p*<0.001, compared with non-treated control, ^#^*p*<0.05, ^##^*p*<0.01 compared with LPS-treated microglia (ANOVA and Newman-Keuls analyses).
